# Development and validation of a risk prediction score for patients with nasopharyngeal carcinoma

**DOI:** 10.1186/s12935-021-02158-6

**Published:** 2021-08-26

**Authors:** Ning Xue, Guoping Ou, Weiguo Ma, Lina Jia, Jiahe Sheng, Qingxia Xu, Yubo Liu, Miaomiao Jia

**Affiliations:** 1 Department of Clinical Laboratory, Affiliated Tumor Hospital of Zhengzhou University, Henan Tumor Hospital, 127 Dongming Road, Zhengzhou, 450000 China; 2 Department of Laboratory Medicine, State Key Laboratory of Oncology in South China, Collaborative Innovation Center for Cancer Medicine, Guangdong Key Laboratory of Nasopharyngeal Carcinoma Diagnosis and Therapy, Sun Yat-Sen University Cancer Center, Guangzhou, 510060 Guangdong China; 3 Department of Ultrasound, State Key Laboratory of Oncology in South China, Collaborative Innovation Center for Cancer Medicine, Sun Yat-Sen University Cancer Center, Guangzhou, 510060 China

**Keywords:** Nasopharyngeal carcinoma, Risk score, Nomogram, Overall survival

## Abstract

**Background:**

We aimed to develop and validate a predictive model for the overall survival (OS) of patients with nasopharyngeal carcinoma (NPC).

**Methods:**

Overall, 519 patients were retrospectively reviewed in this study. In addition, a random forest model was used to identify significant prognostic factors for OS among NPC patients. Then, calibration plot and concordance index (C-index) were utilized to evaluate the predictive accuracy of the nomogram model.

**Results:**

We used a random forest model to select the three most important features, dNLR, HGB and EBV DNA, which were significantly associated with the OS of NPC patients. Furthermore, the C-index of our model for OS were 0.733 (95% CI 0.673 ~ 0.793) and 0.772 (95% CI 0.691 ~ 0.853) in the two cohorts, which was significantly higher than that of the TNM stage, treatment, and EBV DNA. Based on the model risk score, patients were divided into two groups, associated with low-risk and high-risk. Kaplan–Meier curves demonstrated that the two subgroups were significantly associated with OS in the primary cohort, as well as in the validation cohort. The nomogram for OS was established using the risk score, TNM stage and EBV DNA in the two cohorts. The nomogram achieved a higher C-index of 0.783 (95% CI 0.730 ~ 0.836) than that of the risk score model 0.733 (95% CI 0.673 ~ 0.793) in the primary cohort (*P* = 0.005).

**Conclusions:**

The established risk score model and nomogram resulted in more accurate prognostic prediction for individual patient with NPC.

## Background

Nasopharyngeal carcinoma (NPC) is an endemic malignancy characterized by its unique geographic distribution [1]. NPC contributes to a large part of the overall cancer burden among prevalent areas, including southern China, southeast Asia and northern Africa [2, 3]. Radiotherapy is the mainstay treatment for non-metastatic NPC. Chemotherapy combined with radiotherapy is recommended for treatment of advanced NPC [4, 5]. However, the current TNM staging system that is utilized for guidance of the different treatment regimens is insufficient, as many varied clinical outcomes of patients at same stages have been reported [6]. Therefore, there is a need for more accurate indicators to predict prognosis to achieve effective clinical treatment.

Recently, there have been several attempts to improve NPC prognostication through the use of blood molecular biomarkers, including circulating Epstein-Barr virus (EBV) DNA, serum lactate dehydrogenase (LDH) [7], globulin (GLOB), hs-CRP [8], and neutrophil/lymphocyte ratio (NLR) [9]. The infection of EBV is virtually 100% associated with NPC in endemic areas. In addition, the plasma EBV DNA has gradually been used in clinical applications and is considered to be the most attractive potential biomarker to complement TNM staging system [10]. The derived neutrophils to leukocytes ratio (dNLR) has been linked to the inflammatory status and clinical outcomes among several cancers, including NPC [11]. This indicates that dNLR likely has prognostic value and also has the advantage of being inexpensive and easy to calculate. Hemoglobin (HGB) levels have been regarded as important determinants of outcome for a number of cancers treated with radiotherapy, especially gynecological tumors and NPC [12]. However, it remains a challenge to screen and incorporate biomarkers into a new staging system for NPC patients.

A random forest model is an effective classifier as it can predict class of input, and select its most important features [13]. Nomograms have currently been proven to be an effective tool to predict prognosis of patients with cancers, including lung cancer [14], rectal cancer [15], and gastric cancer [16]. In this study, we utilized a random forest model to screen factors related to prognosis of NPC, and incorporate them into a new staging system by establishing a nomogram model.

## Materials and methods

### Patients and clinical characteristic

We retrospectively reviewed the patients with first diagnosed NPC at Sun Yat-Sen University Cancer Center (SYSUCC) between January 2009 and December 2011. Patients included criteria: (1) All patients were pathologically diagnosed as NPC in SYSUCC for the first time. (2) Patients were not any malignancies besides NPC. (3) Patients did not undergo anti-tumor therapy, and data was collected prior to anti-tumor therapy. (4) No patients were infected with hepatitis B virus or hepatitis C virus. (5) Complete records in the database of medical information, and follow-up data. All the patients were classified based on the 8th edition of the AJCC TNM staging guidelines.

We collected information regarding gender, age, family history, smoking, body mass index(BMI), TNM stage, treatment, white blood cells (WBC), neutrophils, lymphocyte, monocyte, platelets, HGB, NLR, neutrophils/(WBC-neutrophils) ratio (dNLR), lymphocyte/monocyte ratio (LMR), platelet/lymphocyte ratio (PLR), systemic immune-inflammation index (SII), total protein (TP), albumin (ALB), GLOB, ALB/GLOB ratio (AGR), C-reactive protein (CRP), CRP/ALB ratio (CAR), apolipoprotein B (APOA), apolipoprotein B (APOB), APOA/APOB ratio (ABR), LDH, high density lipoprotein(HDL), EBV DNA, virus capsid antigen specific immunoglobulin A (VCA-IgA), early antigen immunoglobulin A (EA-IgA), prognostic nutritional index (PNI) and prognostic index (PI). The PNI was calculated based on the following formula: Alb (g/L) + 5 × lymphocyte count × 10^9^/L [17]. The SII was calculated based on this formula: PLR × Neutrophil × 10^9^/L [18]. The PI score was 0 for patients that had CRP levels of 10 mg/L or less, and a WBC count of 11 × 10^9^/L or less. Patients with only one of these abnormalities were given a score of 1, and patients that had an elevation of both CRP and WBC were allocated a score of 2 [19]. The patients’ data were collected prior to any treatment.

### Statistical analysis

Data analyses was carried out using SPSS standard version 20.0 (SPSS, Chicago, USA) and R software version 3.6.1 (http://www.R-project.org). The cut-off values were determined using the R package “survival” and “survminer”. The Kaplan–Meier method was utilized to estimate OS of patients in high-risk and low-risk groups. The concordance index (C-index) was utilized to assess discriminative ability and predictive accuracy of established random forest model and nomogram. The C-index was calculated and compared using the “survcomp” package. The area under the curve (AUC) was computed using the “survivalROC” package. Calibration of the nomogram for 1-, 3-, and 5-year OS was executed via comparison of the predicted survival and observed survival. All statistical tests were two-tailed, and a P value less than 0.05 was considered to be statistically significant.

## Results

### Patients and clinical characteristics

In total, 519 NPC patients were enrolled in this study. All patients were randomly divided into either the primary cohort (n = 363) or the validation cohort (n = 156). The patients’ demographic data and clinical characteristics in both the primary cohort and validation cohort are described in Table 1. In the primary cohort, 209 (57.57%) patients were males and 154 (42.43%) were females. The mean age (SD) of patients in the primary cohort was 46.05 (10.87) years, and the median OS was 51.0 months (interquartile range [IQR]: 42.3–66.7 months). In the validation cohort, 92 (58.97%) patients were males and 64 (41.03%) were females. The mean age (SD) was 46.87 (11.58) years, while the median OS was 50.4 months (IQR: 41.7–66.0 months). In the primary cohort, the 1-, 3-, and 5-year OS rates were 95.0%, 84.0% and 46.8%, respectively. In the validation cohort, the 1-, 3-, and 5-year OS rates were 98.7%, 84.0% and 45.5%, respectively.

**Table 1:** Demographics and clinical characteristics of patients in the primary and validation cohort

Characteristic	Primary cohort	Validation cohort
	n = (363) No. (%) or Mean ± sd	n = (156) No. (%) or Mean ± sd
Gender		
Male	209 (57.57%)	92 (58.97%)
Female	154 (42.43%)	64 (41.03%)
Age (years)	46.05 ± 10.87	46.87 ± 11.58
Smoking		
No	277 (76.31%)	123 (78.85%)
Yes	86 (23.69%)	33 (21.15%)
Family history		
Yes	87 (23.97%)	50 (32.05%)
No	276 (76.03%)	106 (77.95%)
BMI (kg/m^2^)	23.17 ± 6.74	22.96 ± 3.37
TNM stage^a^		
I	11 (3.03%)	6 (3.85%)
II	47 (12.95%)	22 (14.10%)
III	179 (49.31%)	69 (44.23%)
IV	126 (34.71%)	59 (37.82%)
Treatment		
Radiotherapy	300 (82.64%)	129 (82.69%)
Chemotherapy	63 (17.36%)	27 (17.31%)
WBC (10^9^/L)	7.03 ± 3.33	7.04 ± 3.32
Neutrophils (10^9^/L)	4.55 ± 2.87	4.35 ± 2.25
Lymphocyte (10^9^/L)	1.71 ± 0.73	1.65 ± 0.81
Monocyte (10^9^/L)	0.47 ± 0.24	0.43 ± 1.20
Platelet (10^9^/L)	225.02 ± 69.18	214 ± 67.46
HGB (g/L)	136.63 ± 15.84	137.38 ± 15.62
NLR	3.33 ± 3.82	3.34 ± 2.87
dNLR	2.30 ± 2.28	2.48 ± 4.13
LMR	4.67 ± 4.59	4.58 ± 3.49
PLR	157.39 ± 89.41	156.89 ± 86.88
SII	757.39 ± 822.18	719.40 ± 692.30
TP (g/L)	73.28 ± 5.89	75.56 ± 5.24
ALB (g/L)	43.40 ± 3.29	43.17 ± 3.23
GLOB (g/L)	29.88 ± 4.67	29.39 ± 4.50
AGR	1.49 ± 0.24	1.50 ± 0.26
CRP (mg/L)	4.69 ± 9.93	5.42 ± 9.81
CAR	0.11 ± 0.26	0.13 ± 0.27
APOA (g/L)	1.31 ± 0.25	1.32 ± 0.27
APOB (g/L)	0.99 ± 0.24	0.98 ± 0.25
ABR	1.40 ± 0.42	1.42 ± 0.47
LDH (U/L)	174.47 ± 54.26	176.76 ± 119.25
HDL (U/L)	1.23 ± 0.31	1.221 ± 0.31
EBV DNA (copy/mL)		
< 10^3^	167 (48.8%)	72 (40.5%)
10^3^–9999	77 (20.8%)	31 (20.8%)
10^4^–99,999	66 (16.8%)	31 (22.5%)
10^5^–999,999	36 (8.4%)	10 (9.8%)
≥ 10^6^	17 (5.2%)	12 (6.4%)
VCA-IgA		
< 1:80	59 (17.1%)	28 (16.2%)
1:80–1:320	221 (60.1%)	93 (61.3%)
≥ 1:640	83 (22.8%)	35 (22.5%)
EA-IgA		
< 1:10	111 (30.6%)	51 (28.3%)
1:10–1:20	121 (33.3%)	49 (34.7%)
≥ 1:40	131 (36.1%)	56 (37.0%)
PNI	51.94 ± 5.09	51.4 ± 5.17
PI		
0	297 (81.82%)	119 (76.28%)
1	59 (16.25%)	35 (22.44%)
2	7 (1.93%)	2 (1.28%)

^a^TNM stage was classified according to the AJCC 8th TNM staging system

BMI: body mass index; TNM: Tumor Node Metastasis stage; WBC: white blood cell; HGB: hemoglobin; NLR: neutrophil/lymphocyte ratio; dNLR: neutrophil/WBC-neutrophil ratio; LMR: lymphocyte/monocyte ratio; PLR: platelet/lymphocyte ratio; SII: systemic immune-inflammation index; TP: total protein; ALB: albumin; GLOB: globulin; AGR: ALB/GLOB ratio; CRP: C-reactive protein; CAR: C-reactive protein/albumin ratio; APOA: apolipoprotein AI; APOB: apolipoprotein B; ABR: APOA/APOB ratio; LDH: lactic dehydrogenase; HDL: high density lipoprotein; EBV: Epstein-Barr virus; VCA-IgA: viral capsid antigen specific immunoglobulin A; EA-IgA: early antigen immunoglobulin A; PNI: prognostic nutritional index; PI: prognostic index

### Model construction based on clinical characteristics

**Fig. 1:**
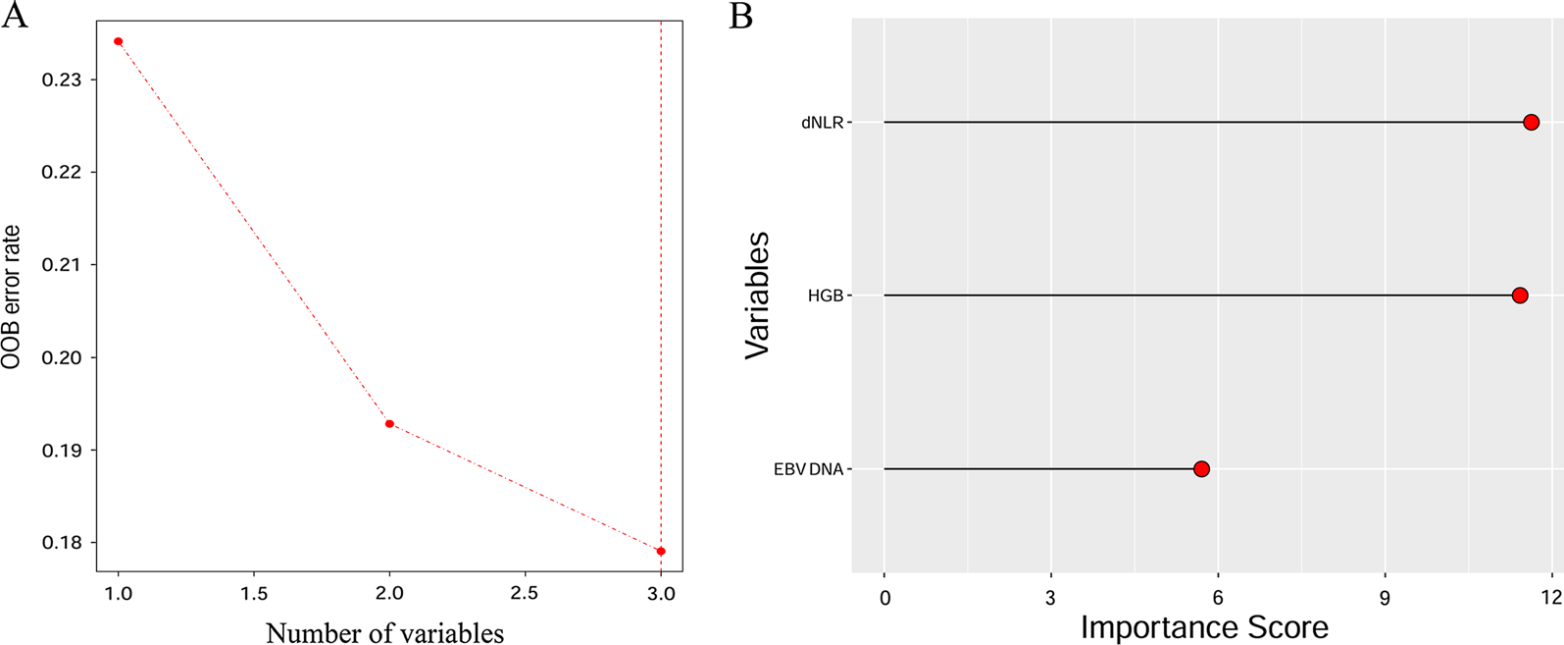
Ranger provides variable importance score for variables for NPC patients in the primary cohort. ‘Out of bag (OOB)’ error rate of top 3 variables in the model (**A**), when probes were included one by one based on their variable importance score ranks (**B**)

**Fig. 2:**
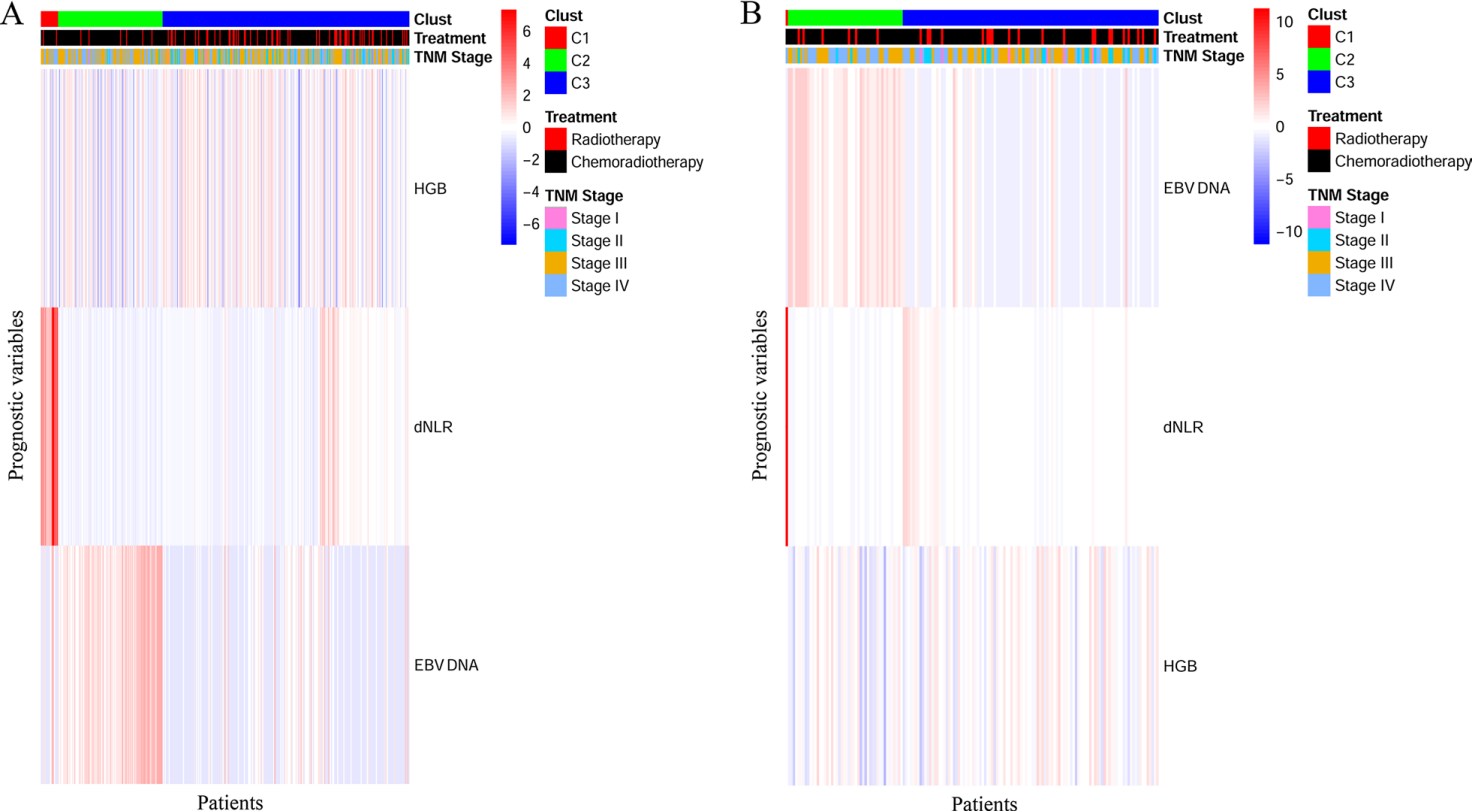
Heatmap were generated by unsupervised hierarchical clustering of 3 features (Y axis) across identified NPC patients on X-axis revealed 3 major image feature patterns in the primary cohort (**A**) and the validation cohort (**B**). The corresponding treatment and TNM stage that the tumor was derived from are shown above the color bars

In the primary cohort, we utilized a random forest model to select the most significant variables from 34 different clinical features. In addition, we used the sliding windows sequential forward feature selection method (SWSFS) to identify important variables by minimizing the ‘out of bag (OOB)’ error rate (Fig. 1A). According to the minimum OOB, three variables including dNLR (HR = 1.14; 95% CI 1.05–1.23; *P* = 9.14 × 10^–4^), HGB (HR = 0.98; 95% CI 0.97–0.99; *P* = 5.24 × 10^–3^) and EBV DNA (HR = 1.59, 95% CI 1.32–1.93, *P* = 1.22 × 10^–6^) were found to be significantly associated with OS among NPC patients (Fig. 1B). Finally, we constructed a risk score model that included dNLR, HGB and EBV DNA. The computational formula of the risk score was as follows: risk score = (0.466 × DNA) + (0.129 × dNLR) −  (0.02 × HGB). The heatmap of NPC samples among the two cohorts are shown in Fig. 2, in which red represents upregulated imaging features and blue represents downregulated imaging features. The three feature clusters (C1–C3) were identified within the heatmap using unsupervised hierarchical clustering of 3 imaging features.

### Model evaluation

**Fig. 3:**
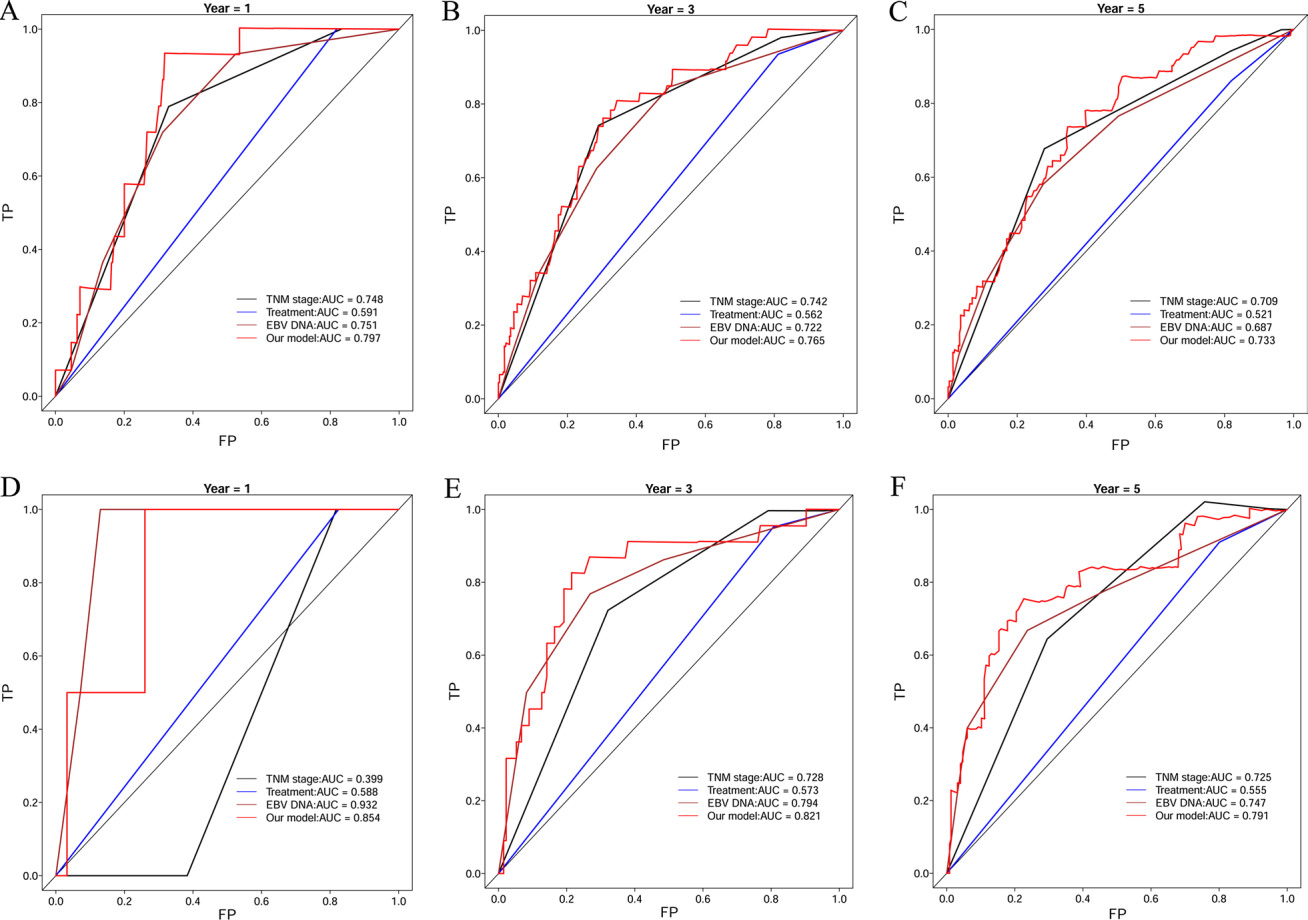
Comparison of AUC among TNM stage, treatment, EBV DNA, and our model in 1-year overall survival (OS), 3-year OS, and 5-year OS in the primary cohort (**A**–**C**) and the validation cohort (**D**–**F**)

**Fig. 4:**
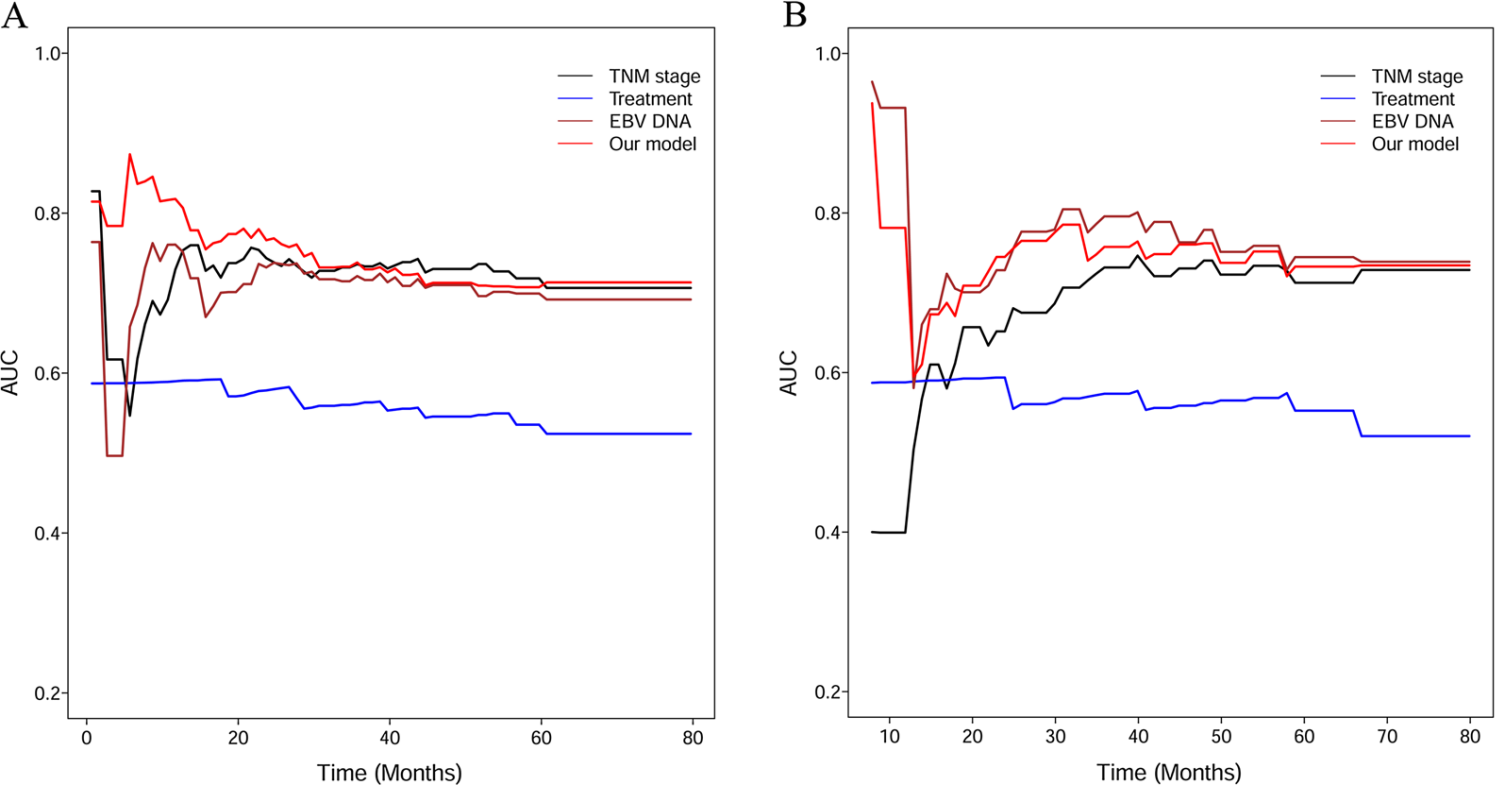
Time-dependent ROC curve for OS in the primary cohort (**A**) and the validation cohort (**B**). ROC: receiving operative characteristics; OS: overall survival

**Fig. 5:**
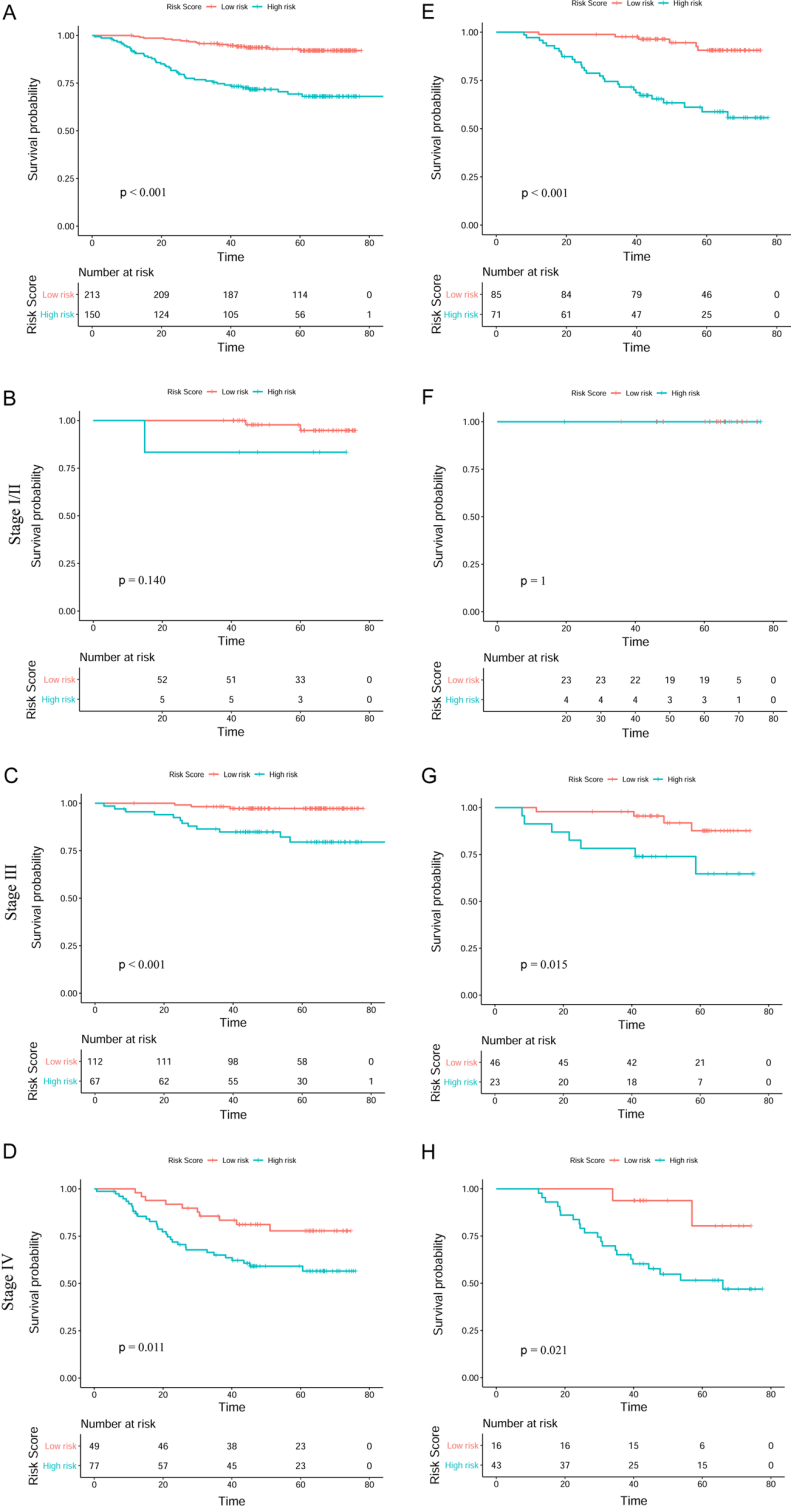
Kaplan–Meier analyses of OS according to the risk score classifier in subgroups of NPC patients in the primary cohort (**A**–**D**) and the validation cohort (**E**, **H**): total patients (**A**, **E**); stage I/II (**B**, **F**); stage III (**C**, **G**); stage IV (**D**, **H**)

ROCs were utilized to evaluate the accuracy of the established risk score model, TNM stage, treatment, and EBV DNA. In the primary cohort, for the 1-year OS (Fig. 3A), the AUC of TNM stage, treatment, EBV DNA and our established model were 0.748, 0.591, 0.751 and 0.797, respectively. Moreover, our model achieved a higher AUC than the TNM stage, treatment, EBV DNA for both the 3-year OS (Fig. 3B) and 5-year OS (Fig. 3C). In the validation cohort, for the 1-year OS (Fig. 3D), the AUC of TNM stage, treatment, EBV DNA, and our established model were 0.399, 0.588, 0.932 and 0.854, respectively. For 3-year and 5-year OS, the AUC of TNM stage, treatment, EBV DNA, and our established model were 0.728, 0.573, 0.794, 0.821 and 0.725, 0.555, 0.747, 0.791, respectively (Fig. 3E, F). The results of a time-dependent ROC curve for OS in the primary (Fig. 4A) and validation cohort (Fig. 4B) demonstrated the AUCs of TNM stage, treatment, EBV DNA and our established model in more detail.

Moreover, we evaluated C-Index of the established model, TNM stage, treatment and EBV DNA to predict of OS in the primary and validation cohort. In the primary cohort, the established model achieved a higher C-index of 0.733(95% CI 0.673–0.793) than the TNM stage (0.712; 95% CI 0.657 ~ 0.768), treatment (0.542; 95% CI 0.505 ~ 0.580) and EBV DNA (0.691; 95% CI 0.626 ~ 0.756). In the validation cohort, the C-index of our model, TNM stage, treatment and EBV DNA were 0.772 (95% CI 0.691 ~ 0.853), 0.699 (95% CI 0.628 ~ 0.770), 0.551 (95% CI 0.503 ~ 0.600), 0.739 (95% CI 0.652 ~ 0.826), respectively (Table 2).

**Table 2:** The C-index of the prognostic model, TNM staging, Treatment, and EBV DNA for prediction of OS in the training cohort and validation cohort

Factors	C-index (95% CI)	*P*
For training cohort		
Our model	0.733 (0.673 ~ 0.793)	
TNM staging	0.712 (0.657 ~ 0.768)	
Treatment	0.542 (0.505 ~ 0.580)	
EBV DNA	0.691 (0.626 ~ 0.756)	
Prognostic model vs TNM staging		0.531
Prognostic model vs Treatment		< 0.001
Prognostic model vs EBV DNA		0.035
For validation cohort		
Our model	0.772 (0.691 ~ 0.853)	
TNM staging	0.699 (0.628 ~ 0.770)	
Treatment	0.551 (0.503 ~ 0.600)	
EBV DNA	0.739 (0.652 ~ 0.826)	
Prognostic model vs TNM staging		0.099
Prognostic model vs Treatment		< 0.001
Prognostic model vs EBV DNA		0.259

C-index: concordance index; CI: confidence interval; P values are calculated based on normal approximation using function rcorrp.cens in Hmisc package

### Performance of the established model in stratifying risk

Based on the computational formula of risk score (0.466 × DNA + 0.129 × dNLR − 0.02 × HGB), NPC patients were subdivided into high risk (risk score ≤ −0.16) and low risk (risk score > −0.16). We used the R package “survival” and “survminer” in order to determine the cut-off values. The optimum cut-off of our model was − 1.46. The results demonstrated that patients with a high-risk score had significantly shorter OS than patients with a low-risk score in the primary cohort (*P* < 0.001; Fig. 5A) and the validation cohort (*P* < 0.001; Fig. 5E). In the primary cohort, the 1-, 3-, and 5-year survival probabilities of the high-risk were 90.0%, 71.3% and 37.3%, respectively. On the other hand, for the low-risk patients, the 1-, 3-, and 5-year survival probabilities were 99.0%, 93.0% and 53.5%, respectively. Meanwhile, in the validation cohort, the 1-, 3-, and 5-year survival probabilities of the high-risk and low-risk patients were 97.2%, 70.4%, 35.2% and 98.8%, 95.3%, 54.1%, respectively (Table 3). Moreover, Kaplan–Meier curves demonstrated that high-risk and low-risk subgroups were significantly correlated with OS outcomes in the primary cohort (Fig. 5C, *P* < 0.001; Fig. 5D, *P* = 0.011), as well as in the validation cohort (Fig. 5G, *P* = 0.015, Fig. 5H, *P* = 0.021) with a respective stage III, and stage IV, with an exception for patients in stage I/II (Fig. 5B, F).

**Table 3:** OS and OS rate in high-risk and low-risk groups according to the established model risk score in the primary and validation cohort

Parameter	Primary cohort			Validation cohort		
	High-risk group	Low-risk Group	Total	High-risk group	Low-risk Group	Total
No. of patients	150	213	363	71	85	156
Median (IQR)	46.5 (27.0–65.9)	61.0 (45.1–68)	51.0 (42.3–66.7)	45.8 (30.5–66.0)	61.2 (45.3–66.0)	50.4 (41.7–66.0)
No. of OS						
1-Year	135 (90.0%)	211 (99.0%)	345 (95.0%)	69 (97.2%)	84 (98.8%)	154 (98.7%)
3-Year	107 (71.3%)	198 (93.0%)	305 (84.0%)	50 (70.4%)	81 (95.3%)	131 (84.0%)
5-Year	56 (37.3%)	114 (53.5%)	170 (46.8%)	25 (35.2%)	46 (54.1%)	71 (45.5%)

OS: overall survival; IQR: interquartile range

### The nomogram for the prediction of OS

**Fig. 6:**
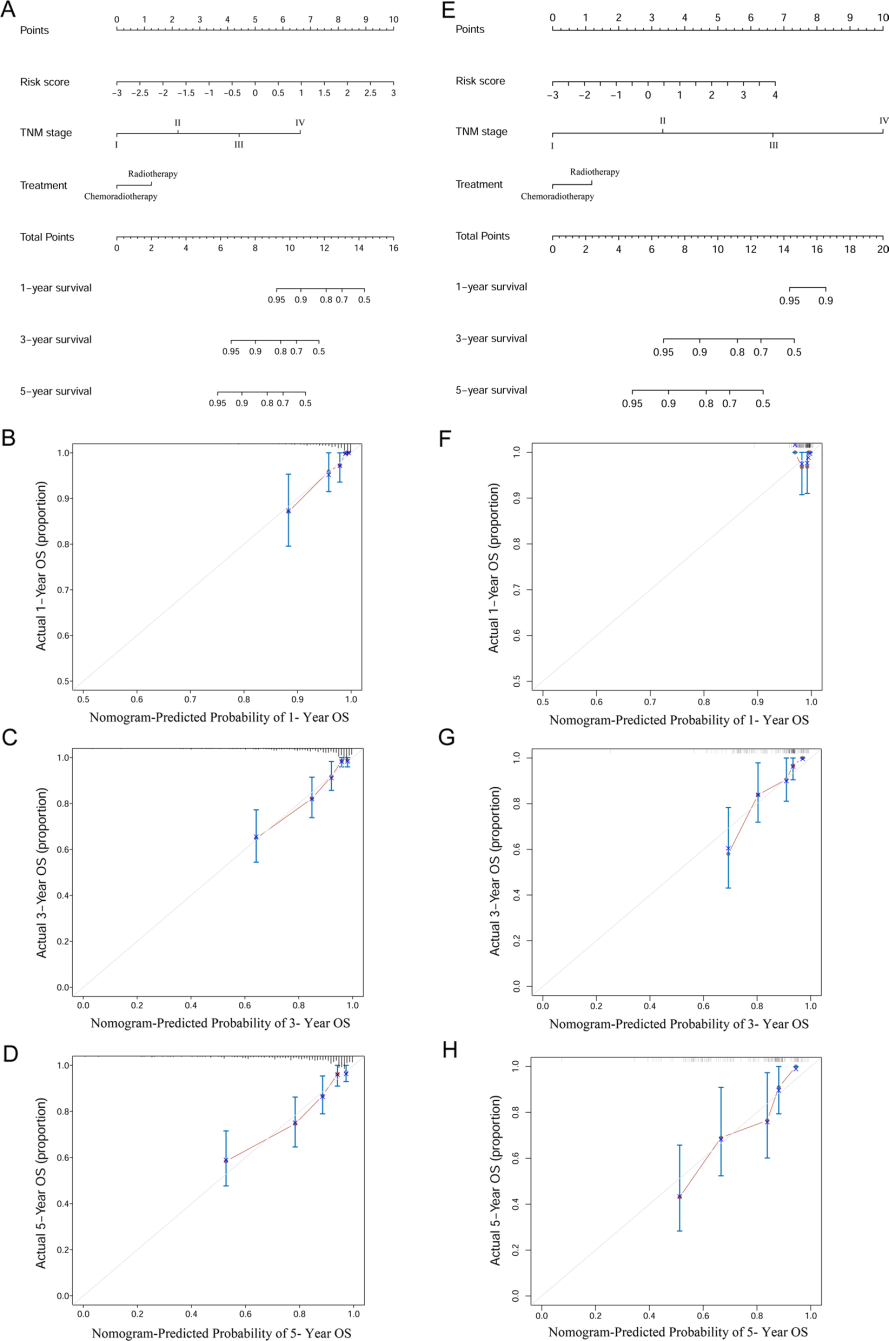
The prognostic model based on risk score, TNM stage and treatment predicting OS in the primary cohort (**A**) and the validation cohort (**E**). The calibration curves for predicting patient OS at 1-, 3-, 5-year in the primary cohort (**B**–**D**) and the validation cohort (**F**–**H**). Total points projected on the bottom scales indicate the probability of 1-, 3-, and 5-year survival

**Fig. 7:**
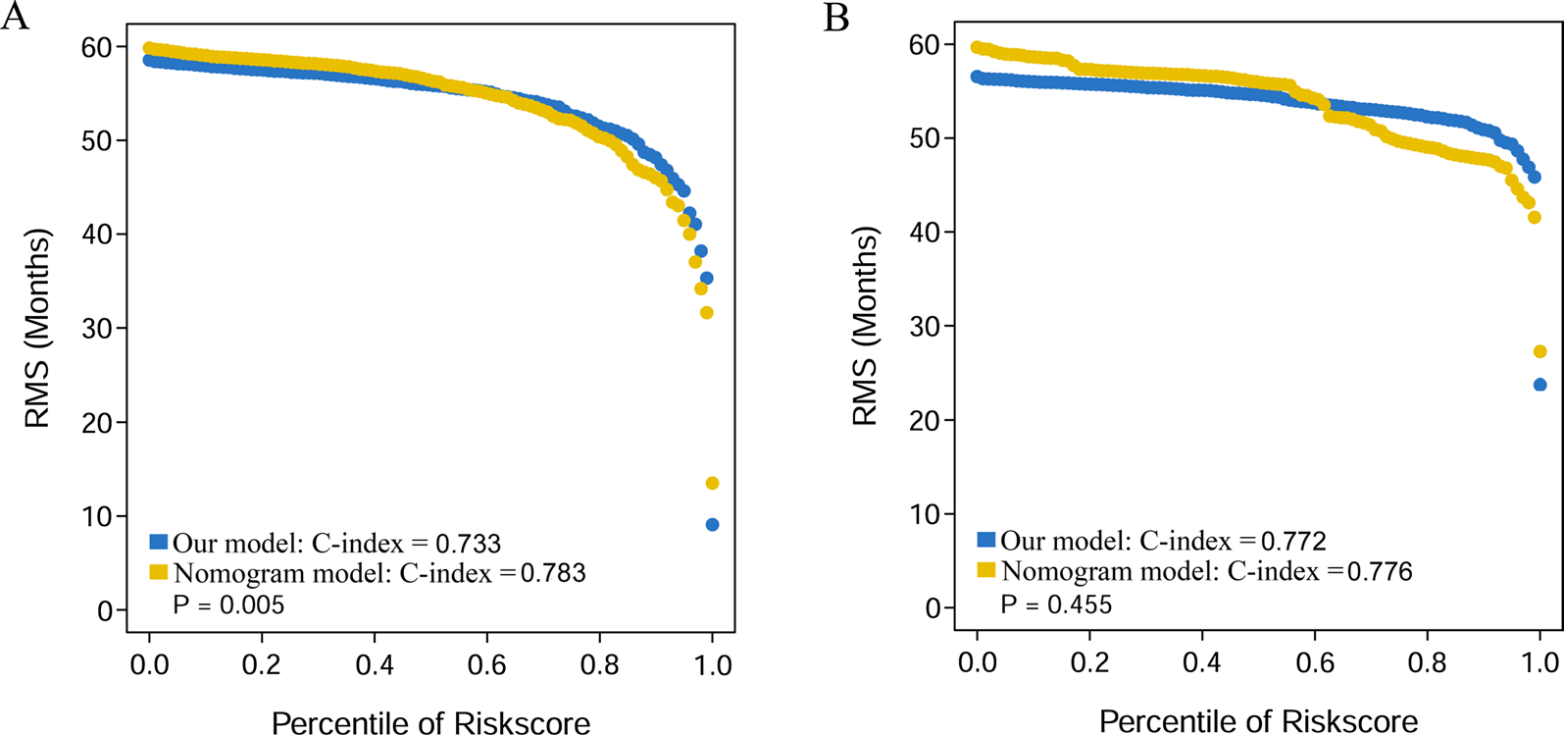
Restricted mean survival (RMS) curves for our model and the nomogram model in the primary cohort (**A**) and the validation cohort (**B**). Each point represents the RMS time of corresponding our model and nomogram model scores

We established a nomogram for OS, which included risk score, TNM stage and EBV DNA in the two cohorts. In the primary cohort, the nomogram model achieved a C-index of 0.783 (95% CI 0.730 ~ 0.836), which was significantly higher than that of the prognostic model 0.733 (95% CI 0.673–0.793, *P* = 0.005) (Figs. 6A, 7A). On the other hand, in the validation cohort, the nomogram model achieved a C-index of 0.776 (95% CI 0.709 ~ 0.844), which was much higher than that of the prognostic model of 0.772 (95% CI 0.691 ~ 0.853, *P* = 0.455) (Figs. 6E, 7B). Calibration curves for probability of survival at 1-, 3-, and 5-years showed optimal agreement between prediction established in the nomogram and actual observation in the primary (Fig. 6 B–D) and validation cohort (Fig. 6F–H). Furthermore, RMS curves demonstrated a larger slope in the primary cohort for nomogram, which indicates superior estimation of survival with nomogram (Fig. 7A, B).

### The correlations among the variables in the nomogram model

**Fig. 8:**
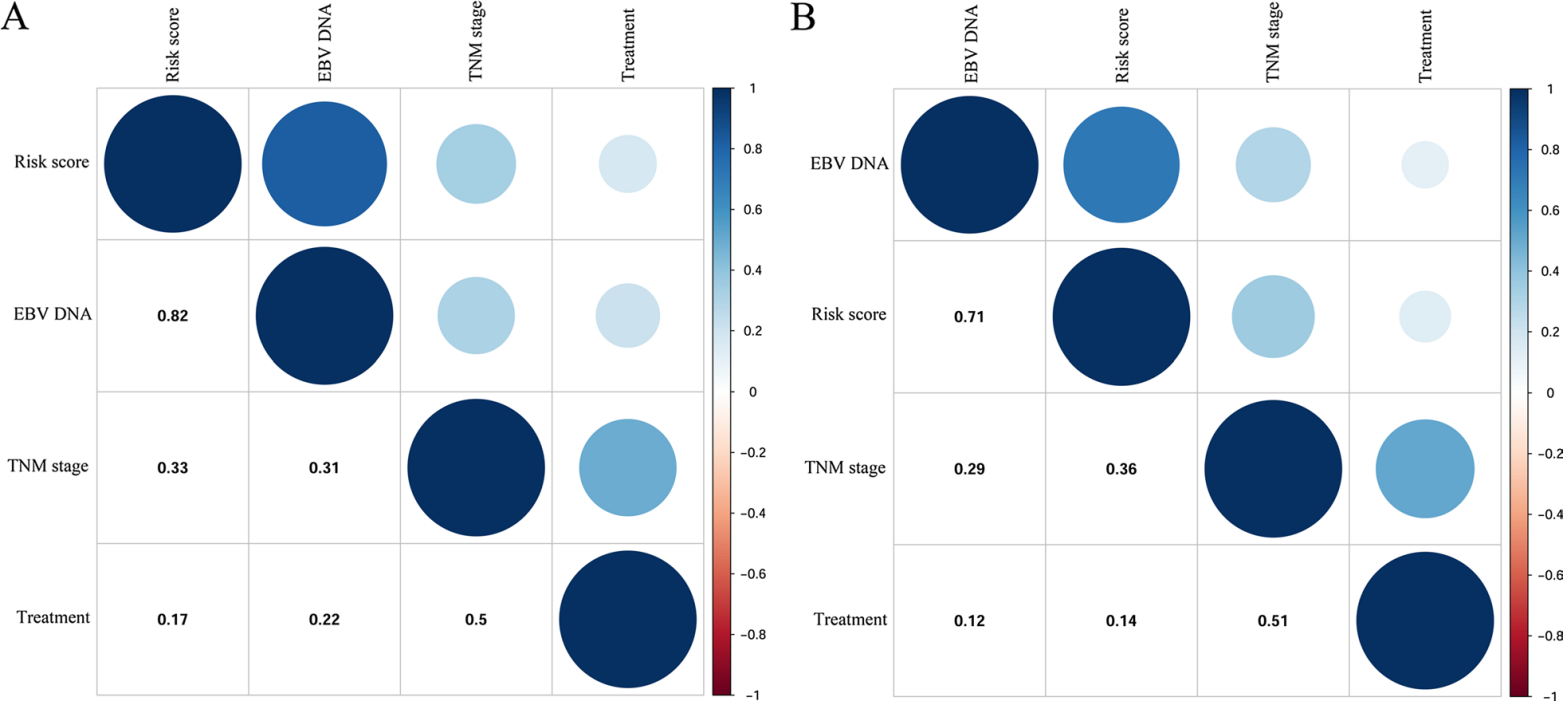
The correlations among the various variables of the nomogram model in the primary cohort (**A**) and the validation cohort (**B**)

The relationship among variables of nomogram model were shown in Fig. 8. In this figure, blue indicated positive correlations, while red indicated negative corrections. Moreover, the correlation coefficients were proportional to color intensity and circle size. In the primary cohort, there was a highly significant between EBV DNA and risk score (Fig. 8A). Meanwhile, treatment was moderately correlated to TNM stage. In the meantime, we were able to get consistent results in the validation group (Fig. 8B).

## Discussion

The TNM stage is commonly utilized to predict prognosis and guide clinical therapeutic regimen across many cancers. This system for NPC was updated and refined to the 8^th^ edition in 2016 [2]. However, this system has several controversies as it is completely based on anatomical extent of cancer, and neglects the biological heterogeneity of NPC patients. Many other important risk factors need to be considered in the current staging systems.

In the present study, we used a random forest model to investigate prognostic value of many clinical factors and selected the most significant ones. We revealed that EBV DNA, dNLR and HGB levels could be used for prediction of NPC prognosis. The established risk score model, which included EBV DNA, dNLR, and HGB have a higher AUC and C-index than TNM stage, treatment, and EBV DNA model in both the primary and validation cohort. Based on risk score, we stratified NPC patients into two subgroups, including high-risk and low-risk, which were significant in OS outcomes. Moreover, according to results from random forest model analysis, we established nomograms that can help predict OS in NPC patients, which integrated risk score, TNM stage, and treatment. The nomogram model showed better predictive accuracy [C-index: 0.783 (95% CI 0.730 ~ 0.836)] than the risk score model [C-index: 0.733 (95% CI 0.673 ~ 0.793) (*P* = 0.005)] in the primary cohort. However, there were no differences in the of C-index between nomogram [C-index: 0.776 (95% CI 0.709 ~ 0.844)] and risk score model [C-index: 0.772 (95% CI 0.691 ~ 0.853) (*P* = 0.455)] in the validation cohort, likely due to our small size of NPC patients in the cohort.

The infection of EBV is common in NPC in endemic areas. Levels of plasma EBV DNA have been shown to be the most attractive potential biomarker to predict prognosis and provide accurate risk stratification in NPC [20]. Intriguingly, a recent prospective screening study that involved 20,174 participants showed that plasma EBV DNA detection was useful to screen for NPC, as it was associated with 97.1% sensitivity and 98.6% specificity. NPC was found to be detected significantly earlier by EBV DNA, with a significantly higher proportion of stage I or II disease than in a historical cohort (71% vs 20%), and had superior 3-year progression-free survival (97% vs. 70%; hazard ratio, 0.10) [21]. However, EBV DNA alone for prognosis has limitations, as the methodology of EBV DNA measurement is not globally standardized and measurement is not routinely available in many medical institutions. Moreover, there is accumulating evidence indicating that inflammation plays an important role in carcinogenesis and tumor proliferation [22]. Studies have reported that inflammation-based markers can be used as potential prognostic factors for many cancers, including CRP, neutrophils, lymphocytes, dNLR and ALB [23–25]. Neutrophils secrete proangiogenic cytokines, which include IL-8, MMP-9, MMP-8, and VEGF, which are known to contribute to tumor angiogenesis and progression [26, 27]. Lymphocytes, especially the CD8+ T cells, which mediated immune response and increased OS of patients with gallbladder cancer [28]. Furthermore, low HGB is a risk factor in cancer patient survival, and HGB level is an important predictor in evaluation and treatment anemia [29, 30]. In our study, we used a random forest model to identify that EBV DNA, dNLR and HGB levels can be used to predict NPC prognosis.

There are several limitations to this study. First, this is a retrospective study, and there may be a selection bias during data collection. Secondly, this is a single-center study with a limited number of NPC patients. Third, this study established models for predicting OS among patients with NPC. However, models of disease-free survival are unknown. Therefore, our next aim is to validate our models on a large-scale with a multi-center study.

## Conclusions

This study established a risk score model based on EBV DNA, dNLR and HGB levels. Compared to TNM stage, treatment and EBV DNA models, risk score model achieved a higher AUC. This easy-to-use scoring prognostic model can provide a more precise estimation for clinicians and patients.

## Data Availability

The datasets analyzed during the current study are not publicly available due to patient privacy concerns, but are available from the corresponding author on reasonable request.

## Abbreviations

BMI: Body mass index
TNM: Tumor Node Metastasis stage
WBC: White blood cell
HGB: Hemoglobin
NLR: Neutrophil/lymphocyte ratio
dNLR: Neutrophil/WBC-neutrophil ratio
LMR: Lymphocyte/monocyte ratio
PLR: Platelet/lymphocyte ratio
SII: Systemic immune-inflammation index
TP: Total protein
ALB: Albumin
GLOB: Globulin
AGR: ALB/GLOB ratio
CRP: C-reactive protein
CAR: C-reactive protein/albumin ratio
APOA: Apolipoprotein AI
APOB: Apolipoprotein B
ABR: APOA/APOB ratio
LDH: Lactic dehydrogenase
HDL: High density lipoprotein
EBV: Epstein-Barr virus
VCA-IgA: Viral capsid antigen specific immunoglobulin A
EA-IgA: Early antigen immunoglobulin A
PNI: Prognostic nutritional index
PI: Prognostic index
